# FAIRsoft—a practical implementation of FAIR principles for research software

**DOI:** 10.1093/bioinformatics/btae464

**Published:** 2024-07-22

**Authors:** Eva Martín del Pico, Josep Lluís Gelpí, Salvador Capella-Gutierrez

**Affiliations:** Barcelona Supercomputing Center (BSC), 08034 Barcelona, Spain; Barcelona Supercomputing Center (BSC), 08034 Barcelona, Spain; Biochemistry and Molecular Biomedicine Department, University of Barcelona, 08028 Barcelona, Spain; Barcelona Supercomputing Center (BSC), 08034 Barcelona, Spain

## Abstract

**Motivation:**

Software plays a crucial and growing role in research. Unfortunately, the computational component in Life Sciences research is often challenging to reproduce and verify. It could be undocumented, opaque, contain unknown errors that affect the outcome, or be directly unavailable and impossible to use for others. These issues are detrimental to the overall quality of scientific research. One step to address this problem is the formulation of principles that research software in the domain should meet to ensure its quality and sustainability, resembling the FAIR (findable, accessible, interoperable, and reusable) data principles.

**Results:**

We present here a comprehensive series of quantitative indicators based on a pragmatic interpretation of the FAIR Principles and their implementation on OpenEBench, ELIXIR’s open platform providing both support for scientific benchmarking and an active observatory of quality-related features for Life Sciences research software. The results serve to understand the current practices around research software quality-related features and provide objective indications for improving them.

**Availability and implementation:**

Software metadata, from 11 different sources, collected, integrated, and analysed in the context of this manuscript are available at https://doi.org/10.5281/zenodo.7311067. Code used for software metadata retrieval and processing is available in the following repository: https://gitlab.bsc.es/inb/elixir/software-observatory/FAIRsoft_ETL.

## 1 Introduction

Software is crucial in contemporary scientific research (Howison *et al.* 2015). As one of the central concepts of this work, research software is understood as any software component used as part of the scientific discovery process or the direct product of the research activity. This dependence on computational technologies is especially strong in research based on computational simulations and data-driven science (Schindler *et al.* 2022). This requires multidisciplinary teams of scientists and engineers collaborating, often in a distributed manner, to provide the needed computational methods and infrastructure to access, store and process data generated by experiments and simulations. In a survey conducted by Hannay *et al.* (2009) to measure the extent to which research depended on computational technologies, 91% of participants said using scientific software was important for their research, and 84% stated that developing scientific software was important or very important for their research.

Unfortunately, despite its relevance, research software is not required to meet the requirements that are usually a must for other scientific methods: being peer-reviewed, being reproducible and allowing one to build upon other’s work (Morin *et al.* 2012, Jiménez *et al.* 2017). Consequently, the research’s computational component is often impossible to reproduce and/or verify (Allison *et al.* 2016). It may even contain errors that affect the outcome of Soergel (2014), is opaque or directly unavailable or can be hard to use by non-developers. These, among other issues, are detrimental to the integrity and reliability of scientific research. As Chen et al. (2019) argue, based on their experience in the high-energy physics community, to tackle this problem:

Services and tools should be developed with the idea of meshing seamlessly with existing research procedures, encouraging the pursuit of reusability as a natural part of researchers’ daily work […]. In this way, the generated research products are more likely to be useful when shared openly

The recent publication of the joint work by the Research Data Alliance (RDA), Research Software Alliance (ReSA) and FORCE11 (Barker *et al.* 2022), introducing the first comprehensive attempt to translate the FAIR (findable, accessible, interoperable, and reusable) principles for research software, following on an initial work carried in Lamprecht *et al.* (2020), represents an important step to enhance software quality and sustainability. This effort was made after successfully applying the FAIR data principles (Wilkinson *et al.* 2016) to scholarly data affected by similar issues, namely the great difficulty of sharing and accessibility.

We present FAIRsoft, our effort to assess elements that contribute to enhancing the research software quality using a FAIR-like framework. Although most conclusions are generally valid, FAIRsoft evaluation is centred in the Life Sciences domains. This effort will become part of OpenEBench (Capella-Gutierrez *et al.* 2017) (https://openebench.bsc.es/), the ELIXIR (https://elixir-europe.org/) open data platform to support the technical monitoring and scientific benchmarking of research software. To be able to do that, we propose a series of measurable indicators based on a pragmatic interpretation of the FAIR principles. Here, we propose an initial scoring system to weigh the different indicators and offer a quantitative assessment of FAIRness. To evaluate the approach’s feasibility, we have used the OpenEBench technical monitoring platform, containing metadata for over 43 000 Life Sciences tools from various sources. The results serve to understand the current practices around research software quality-related features. They could contribute to the community-led discussion on implementing automated indicators and dedicated efforts to improve those practices. Periodic assessment of those major patterns and their public availability through the Software Quality Observatory in OpenEBench could contribute to understanding cultural changes across researchers that code and professional research software engineers.

## 2 Rendering FAIR principles for research software into measurable indicators

The major difference between the general FAIR data principles and the current efforts for translating, consolidating, and proposing dedicated FAIR principles for research software lies in the dynamic nature of software. Research software shares many properties with other digital objects, e.g. research data. However, the possibility of using research software to carry on specific tasks, i.e. being executable, makes it different from the rest. Considering this particular behaviour, we analysed the FAIR principles for research software to formulate a strategy to consider the feasibility of applying and implementing a quantitative evaluation for such principles.

**Figure 1.**
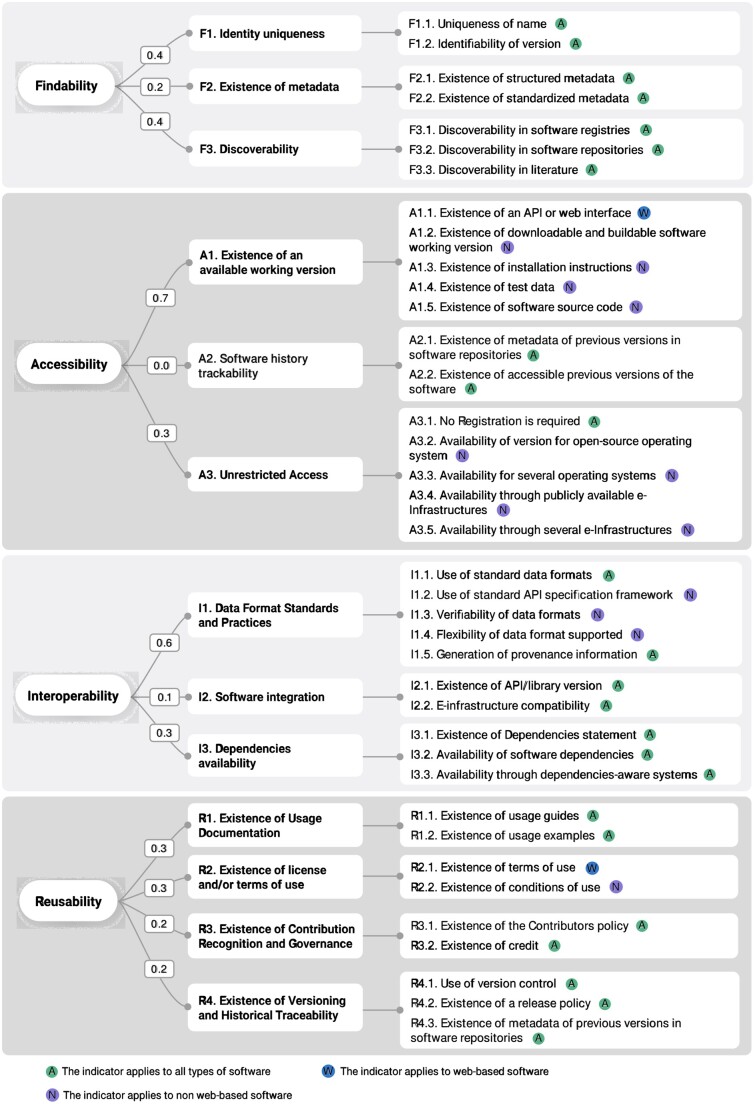
FAIRsoft indicators. Indicators proposed for the four FAIR principles applied to Research Software. Each high-level indicator is linked to specific low-level criteria. Greyed out indicators represent unimplemented features, and icons denote software type applicability. Indicator weights are shown on connecting arrows, influencing total FAIRness scores (low-level indicator weights not displayed)

While developing FAIRSoft, Barker *et al.* (2022) presented the first application of FAIR principles to research software in general, building on previous work carried out in the Life Sciences domain (Lamprecht *et al.* 2020). Their approach proposed a set of indicators, namely FAIR4RS. As expected, this initiative shares most of FAIRSoft interpretations and indicators (Supplementary Table S1 shows the four indicators proposed by FAIR4RS that are not covered by FAIRsoft). However, FAIR4RS is formalized abstractly and does not aim to obtain quantitative assessments. On the contrary, in this work, we wish to generate a set of measurable indicators to allow a useful evaluation of the elements enhancing the quality of research software (see Fig. 1) and provide guidance to developers on how to improve them.

In the context of FAIRsoft, we followed a two-step approach. In the first step, we derived a number of requirements that software must fulfil to be *Findable*, *Accessible*, *Interoperable*, and *Reusable*, respectively. We call these properties high-level indicators. Most of the high-level indicators coincide with FAIR4RS, like ‘A2. Software history trackability’ (Fig. 1, *Accessibility*) or ‘R2. Existence of license and/or terms of use’ (Fig. 1, *Reusability*). However, other high-level indicators are not explicitly found in previous works, like ‘I2. Software integration’. Four indicators in Barker et al. (2022) have no straightforward equivalent among ours, although they are still implicitly covered by other indicators in this work. This is the case of ‘I2. Software includes qualified references to other objects’. We consider any digital object necessary for the correct execution of software as input data. This is consistent with the broadly used EDAM ontology (Ison *et al.* 2013) for annotating research software in Life Sciences. Thus, this indicator is covered in our high-level indicator ‘I1. Data format standards and practices’. Finally, one of our interoperability principles (I3. Dependencies availability) is considered as part of the reusability principles by the community effort (Barker *et al.* 2022) (‘R2. Software includes qualified references to other software’). This difference illustrates the difficulties in translating and adapting the interoperability aspects of research software. In this context, interoperability stands for how the software operates in a given environment, e.g. when it is executed. Another difference in the interoperability domain includes references to non-digital objects, which may have a digital presence. Although conceptually necessary, we did not include them as the automated validation of such relationships would be limited to identifying their existence with little insight into the actual interoperability. Supplementary Table S1 shows a more detailed description of the differences between our guiding principles and associated indicators and the principles proposed by FAIR4RS. As presented, many of those differences are minimal and are driven by the practical implementation of the associated indicators (see specific comments in Supplementary Table S1). The second step takes us from high-level indicators to the desired degree of granularity by generating low-level indicators. A low-level indicator is one condition, often a present/absent value, that contributes to research software meeting a high-level one. Additionally, a low-level indicator is associated with a well-defined evaluation procedure to allow their direct implementation. For example, to fulfil the high-level indicator ‘F3. Discoverability’, software should be (i) included in any main software registry, e.g. bio.tools (https://bio.tools) (Ison *et al.* 2016), (ii) available in any of the major software repositories, e.g. GitHub (https://github.com), GitLab (https://about.gitlab.com), SourceForge (https://sourceforge.net), or (iii) found in specialized literature services, e.g. EuropePMC (https://europepmc.org/) (The Europe PMC Consortium, 2015), PubMed (https://pubmed.ncbi.nlm.nih.gov) (Sayers *et al.* 2022), Journals Site, bioRxiv (https://www.biorxiv.org). Thus, F3 can be evaluated using the appropriate search engines in such registries or repositories. To summarize, these low-level indicators can be pictured as conforming checklists to fulfil the requirements of high-level indicators. We aimed low-level indicators to be straightforward enough to be easily followed by a developer interested in assessing the FAIRness of a particular software, and easily evaluated by an external, possibly automated, monitoring system. To reduce possible ambiguities in their application, low-level indicators were defined following the FAIR metrics working group scheme (Wilkinson *et al.* 2018) that includes explicit answers to: what is being measured, why we should measure it and how we measure it (see Supplementary Figs S1–S4 for full details on high- and low-level indicators hierarchy).

While high-level indicators apply to all kinds of software, not all low-level indicators do so since the requirements to fulfil a high-level indicator may depend on the type of software. For instance, the conditions for a web application to be accessible differ from a command-line tool. To keep our set of indicators as simple as possible, we only distinguish between what we consider the minimum number of software categories necessary for our purpose: ‘web’ and ‘non-web’ tools. Consequently, each low-level indicator can apply exclusively to ‘web’, exclusively to ‘non-web’ or to both types of tools (see Fig. 1 for further details).

As the translation of the FAIR principles for research software is an iterative effort, any indicators associated with them will naturally evolve following a community-driven effort. As these efforts mature, better indicators can be derived over time, with the focus being on their automated measurement. Altogether, it will eventually provide a temporal perspective on the evolution of these efforts and their impact on the broader research community.

### 2.1 Proposal for a FAIRsoft scoring system

To translate the fulfilment of low-level indicators into an objective evaluation, low-level indicators were assigned a weight that encapsulates their relative relevance in the context of the associated high-level indicator. Similarly, each high-level indicator was assigned a weight that summarizes its relevance for the FAIR principle it was associated with. This allowed us to accumulate scores, ranging from 0 to 1, for high-level indicators and principles of an instance. Supplementary Figure S5 offers a practical example of how high- and low-level indicators are measured for a given software, e.g. trimAl v1.4.1 Weights for all indicators were assigned to reflect their relevance and dependencies following the initial data collected. Indeed, selecting such weights is necessarily arbitrary and subjected to further revision as the principles evolve. Supplementary Figures S1–S4 summarize the details of the FAIRsoft scoring system, including the methodology used in their evaluation from the metadata available in OpenEBench. It is important to clarify that the scores we propose here do not aim to be an absolute measurement of FAIRness but a summary value that captures the level of fulfilment of a comprehensive set of indicators. An appropriate assessment of FAIRness would require examining each item with greater detail. Nonetheless, quantitative FAIRsoft scores constitute a valuable tool for exploring software quality-related features, especially in the context of a population of tools. This effort allows us to identify general trends and propose targeted actions to improve the overall research software quality (see examples below).

In our approach to research software FAIRness, we faced the need to differentiate between two information levels for the same software resource: canonical entries and instances. We use the term *canonical tool* to refer to an abstract notion of a given research software as a computational solution that has been implemented and given an identity name by its author/s. Then, software resources can be materialized in different ways in terms of how users interact with it, e.g. command-line applications, web applications, libraries; availability, e.g. desktop and/or web applications; or differences in the code that are not big enough to justify considering them as distinct tools, e.g. different versions of the same software. Each of these constitutes an *instance* of the same canonical tool. The natural way of assessing software FAIRness requires the analysis of such instances, as only a subset of the indicators can be common and hence assigned to the canonical entry.

## 3 Results

Benchmarking consists of measuring the performance of research software from a technical, functional, and/or scientific perspective. Indeed benchmarking efforts conform to a highly diverse scenario that helps developers and researchers to evaluate their tools and choose the best-fitted one for their scientific needs. However, due to the increasing amount of resources and communities, large-scale efforts for developing, maintaining, and extending centralized infrastructures that support those community efforts are essential. In this context, OpenEBench, an initiative developed within ELIXIR, aims to provide a permanent platform to support benchmarking in Life Sciences. OpenEBench provides, on the one hand, support to scientific communities to perform a critical evaluation of the scientific performance of methods and tools in specific domains (Altenhoff *et al.* 2020, Harrow *et al.* 2021, Petrillo *et al.* 2021). On the other hand, OpenEBench maintains a Tools Monitoring section, holding updated metadata for bioinformatics research software, extracted from data sources like bio.tools, Bioconda (https://bioconda.github.io) (Grüning *et al.* 2018), Galaxy (https://usegalaxy.eu) (Afgan *et al.* 2016) among others (see below), and provides live analysis of indicators regarding availability, documentation or licence usage, among others. One of the ultimate goals of OpenEBench is to provide an observatory of the quality of the Life Sciences research software, which is able to provide an overall view of the field, to support the development of best practices, and measure its progressive adoption.

### 3.1 Retrieval, transformation, and integration of metadata

Automated evaluation of indicators for large amounts of software entries and the subsequent assessment of their quality requires the development of specific data and metadata retrieval, transformation, and integration mechanisms. For each tool, FAIRsoft indicators can be measured using, in most cases, metadata from more than one reference resource, which must be accessible and findable by any user in order to be valid. These resources include software registries and repositories, e-Infrastructures, software homepages, and journal publications. Importantly, those data and metadata resources can be extended to increase the indicators coverage for individual entries.

**Figure 2.**
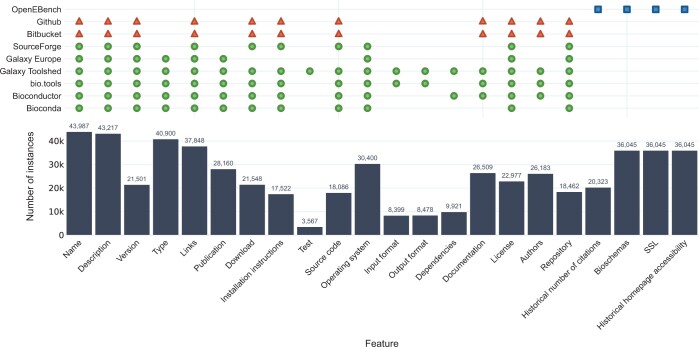
Features obtained from the different software metadata sources and the total number of instances for which each feature exists in the dataset. Green circles denote features obtained from primary metadata sources, while red triangles represent features obtained from secondary sources. Blue squares depict features available in OpenEBench after combining primary and secondary metadata sources. Bars indicate the actual number of retrieved metadata items. Variation of the amounts is a consequence of the lack of completeness of metadata annotation

We aim to assess the quality of research software in Life Sciences. At this stage, we have integrated metadata from various resources. Bio.tools, Bioconda, Bioconductor (Gentleman *et al.* 2004), Galaxy ToolShed (Blankenberg *et al.* 2014), SourceForge, and Galaxy Europe (Afgan *et al.* 2016) constitute the primary sources used to discover tools and retrieve an initial collection of metadata (Supplementary Table S2). In addition, secondary sources were mined to enrich tool entries obtained from the initial sources. These are GitHub, Bitbucket (https://bitbucket.org), OpenEBench, PubMed, Europe PMC, and Wikidata (https://wikidata.org) (Vrandečić and Krötzsch, 2014) (Supplementary Table S3). Each resource provided information that was either structured or unstructured and retrievable through different means. As a consequence, data required different degrees of processing before being integrated into the framework (Supplementary Fig. S6). In brief, data coming from primary sources were processed to identify commonalities and differences. Shared names, software IDs, and reference URLs were used as criteria to combine individual records from different sources. Unique metadata and data were used to decide whether one or more different instances were needed for a given resource. Then, data from different sources was restructured to fit a common data model and integrated by instance (using a common name-type pair as the main identifier), leading to a set of 43 987 unique instances. The number of tools with available metadata varied greatly among resources, as well as the type of information they provided. Indeed, the majority of instances, 36 075 out of the total 43 987, were enriched with metadata from more than one source (Supplementary Fig. S7). See Figure 2 for a list of features considered in our common data model and their coverage by the different resources.

The type of information we retrieved more successfully are version and links, usage of bioschemas (Gray *et al.* 2017) in the homepage, the existence of an SSL certificate on the homepage and historical homepage accessibility and description. On the other hand, test data could only be retrieved for instances present in the Galaxy Toolshed, which represents just 8.1% of the total. Information on dependencies, repositories, and input and output data formats are underrepresented in our set of instances metadata as well.

For each indicator, an algorithm was further designed and implemented to decide whether that indicator was being fulfilled or not (Supplementary Tables S1–S4). Automated measurement of indicators is a requirement for two main reasons. First, it is important to be able to reproduce the analysis at any point in time. Second, periodic execution of those algorithms will allow us to understand how research software evolves over time and capture newly registered tools. Some of the initially proposed indicators were not computed, given the lack of appropriate (meta)data that can be inferred automatically. Then, a score of FAIRness was calculated for each instance, combining the different measured indicators for the four general principles. As not all indicators are equally relevant, a weighting scheme was designed and implemented to reflect the varying importance of individual indicators (Supplementary Tables S1–S4).

### 3.2 FAIRness analysis

**Figure 3.**
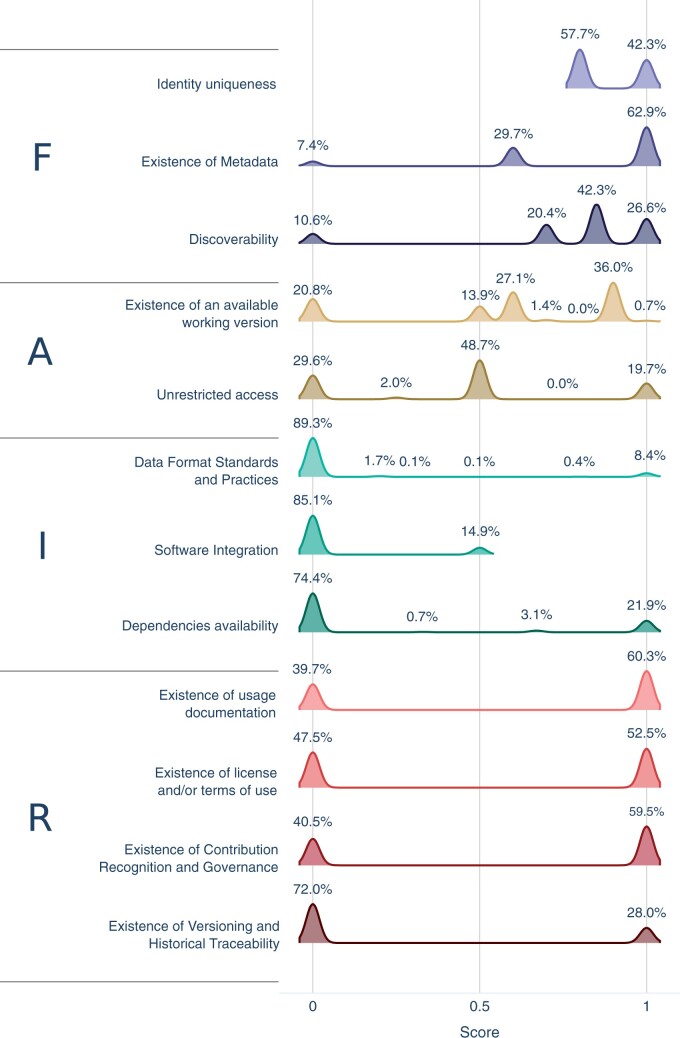
High-level indicators scores of instances. For each high-level indicator, possible scores are determined by the way in which low-level indicators are weighed to compute the high-level score. Each possible score is labelled with the percentage of instances scoring it. Although scores are discrete values, they are shown as density plots for clarity

Once the metadata is integrated and consolidated into the OpenEBench platform, FAIRsoft indicators can be computed individually for the 43 987 tool instances. This process is performed periodically to have the most up-to-date figures about the analysed research software. Then, individual scores for the 12 high-level and their associated low-level indicators (see Supplementary Tables S1–S4) can be summarized to understand the FAIRness level of the evaluated collection. In general terms, research software in Life Sciences is highly *Findable*, moderately *Accessible* and *Reusable*, and barely *Interoperable*, according to our indicators. It is important to note that software that cannot be easily identified and found in reference resources, e.g. registries and repositories, is likely to be overlooked in this analysis. Results of such analysis provide, on one hand, a global picture of FAIRness in the analysed set of tools (Fig. 3), and, on the other hand, a detailed summary of specific aspects of software quality.

**Figure 4.**
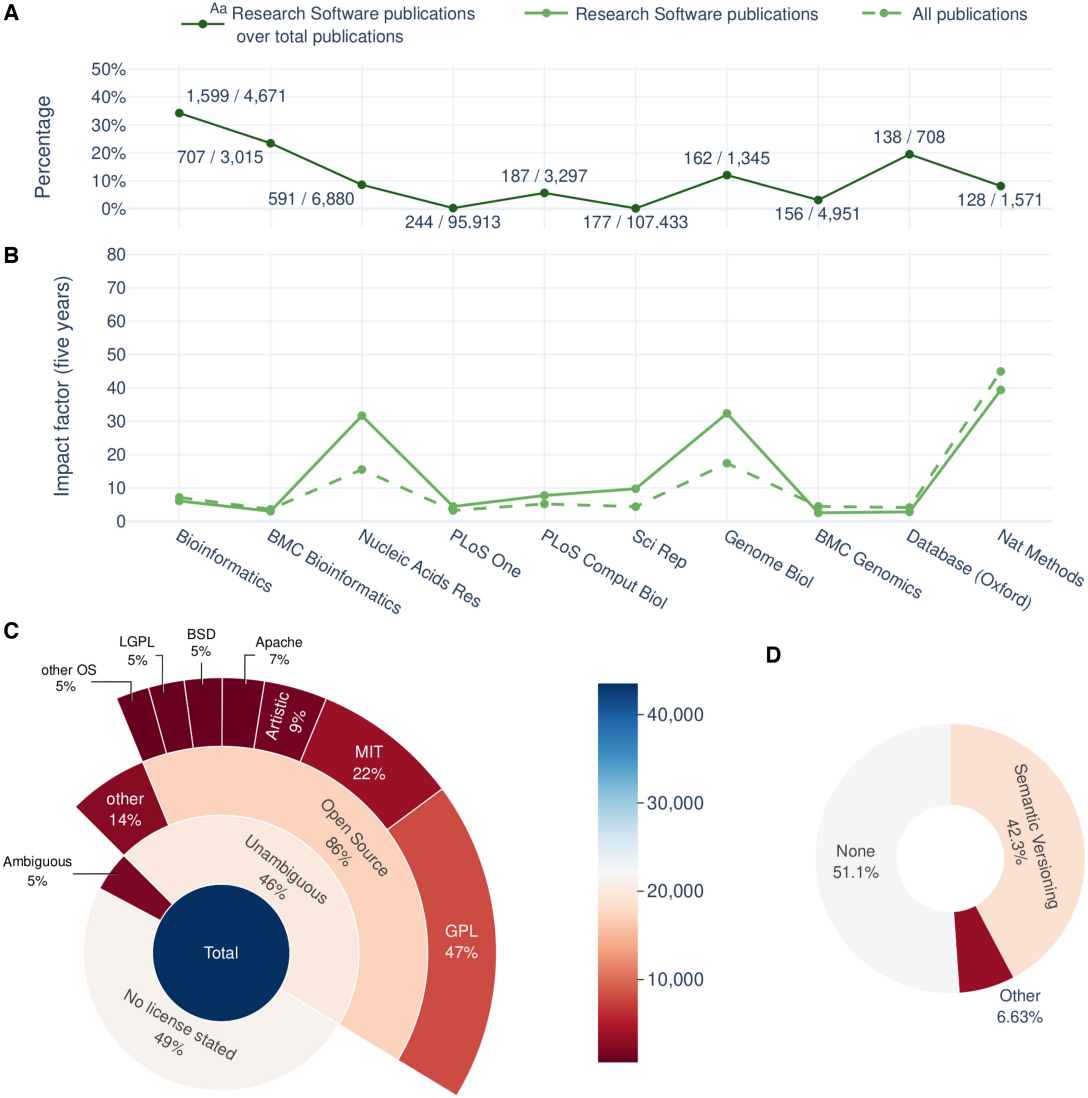
Relevant factors of research software analysed. (A) Journals with the highest rates of research software publications and percentage represented by those publications over the total publications in the last 5 years. The annotation of each point shows the absolute number of research software publications over the absolute number of total publications. (B) Impact factor of research software publications by journal. Five years of journals’ impact factor, extracted from reliable sources, and impact of research software publications, calculated using data extracted from OpenEBench. The journals analysed are the ones that have published the most research software papers in the last 5 years. (C) Licencing. The proportion of licencing statements, ambiguous and unambiguous licencing, Open Source Licences among unambiguous licences, and main OS licence families. Licencing is considered ambiguous when different sources attribute a different licence to the same instance and unambiguous otherwise. (D) Versioning scheme. The proportion of instances without versioning, versioned using Semantic Versioning, and versioned using other versioning schemes

Results for the four general FAIR principles are heterogeneous. However, patterns can be easily identified among them. For instance, indicator scores for the *Findability* of research software are naturally higher than others, as it is nearly impossible to measure any indicator for software that cannot be found. A lack of structured metadata (*F2.1: Existence of structured metadata*) is the main reason for those cases with a lower findability score. Special mention should be given to the existence of an associated publication respective to a given Software (*F3.3: Discoverability in literature*). Any software described in an indexed peer-reviewed article heavily increases its *Findability* and *Reusability*. A scientific publication unequivocally associated with a piece of research software provides a reliable, unique, persistent, and global identifier, the publication Digital Object Identifier (DOI). Scientific publications generally offer a careful description of the software, often including the domain of application and usage and details about accessibility and availability, including link/s to a repository in the best-case scenario. Moreover, a publication can serve as the reference to credit authors of a software and is actually the most common way to cite software in research publications (Howison and Bullard, 2016). This is of major relevance in the current circumstances of research software being under-cited and the absence of standards for software citation (Howison and Bullard, 2016, Park and Wolfram, 2019). We found a high proportion of published software in our data (20,608/43,987; 47.0%). This proportion is bigger than the usage of version control repositories (28.0%, *R4.1 Use of version control*) and comparable to software licencing (52.5%, *R2: Existence of licences and/or terms of use*). If we consider unique publications, there are 16 994 ones published across 532 different journals, of which OUP *Bioinformatics*, *BMC Bioinformatics*, and *Nucleic Acid Research* are the most common ones. Journals publishing the highest number of software manuscripts with associated software are shown in Fig. 4A. Although none of them is exclusively devoted to software, software publications can represent a significant proportion of the total publications of the journal in some cases, e.g., 35.3% in OUP *Bioinformatics*. Regarding the impact of software publications in these 10 journals (Fig. 4B), we found that, in general, the average impact factor of software publications is either equal or even higher than the one for the entire journal.

The actual usability of the software includes indicators from two principles: *Accessibility* and *Reusability*. *Accessibility* is considered here in terms of gaining access to the software across different means and using it either by just downloading it, e.g. a binary; building it from the source code, or directly using it through a web-based application. *Reusability* refers to the conditions of the software usage. Although the aim of research software is to be used, only 0.7% of analysed tools (Fig. 3) show optimal scores for *Accessibility* (*A1: Existence of available working version*). Most deficiencies arise from the unavailability of test data. Interestingly, nearly 70.4% of the instances have no use restrictions (*A3: Unrestricted access*), confirming the increasing trend for adopting the Open Science paradigm, at least in the Life Sciences domain. *Reusability* varies greatly, with a wide range of scores due to the diversity of forms in which software exists and the many factors determining it. However, some factors stand out, namely the absence of licences (*R2: Existence of licence and/or terms of use*). Slightly over 49% of the instances lacked a licence statement (Fig. 4C). For the remaining ones, we find coherence between data sources regarding licence families, i.e. GPL, LGPL, MIT; in the vast majority of cases, with only a few marginal cases of two or more different licence families stated (5%). The latter can be due to different versions of tools actually having differing licences or due to erroneous metadata. Regarding instances with coherent stated licences, 86% were identifiable open-source ones. Even when a piece of research software is explicitly mentioned in a research publication, the specific version used is still sometimes left unspecified (Howison and Bullard, 2016), severely hindering attempts to reuse it or to reproduce published results. For this reason, ‘Specificity’ is one of the Software Citation Principles (Smith *et al.* 2016). The usage of a versioning scheme in terms of version identification (*F1.2: Identifiability of version*) or provenance (*R4.1: Use of version control*) (Fig. 4D) emerges as a key factor to allow the proper use of the different versions of an evolving artefact such as a piece of software and, thus, guaranteeing both its *Findability* and *Reusability*. We found that only 48.9% of the software analysed had a version statement, of which 86.5% followed the Semantic Versioning scheme while the remaining 13.5% identified versions did so after the release date or following unspecified conventions.

Associated indicators for Interoperability (Fig. 3) show that this is the principle with the lowest scores. It does not necessarily mean that research software is not interoperable *per se*, but it points out important aspects to consider regarding the evaluation of interoperability. On one hand, there is abundant literature on what software interoperability is and how it can be measured in terms of working with other research software as part of analytical workflows (Goble *et al.* 2020), or in terms of interoperating with underlying software components such as software libraries. On the other hand, no agreed standards to capture and represent interoperability exist. This situation makes it difficult to have objective indicators that can be measured automatically for massive collections of tools. Aspects like documentation of formats (*I1: Data Format Standards and Practices*) and dependencies (*I3: Dependencies availability*) are the most straightforward to measure. However, this information is only structured when it should be machine-readable as in package repositories, while when obtained from the documentation, it may not be complete or even described. The real ability to interoperate (*I2: Software Integration*) is often un-documented except for software libraries, APIs or when software is described as part of a pipeline. The latter requires considering other sources of data and metadata, which are not the main focus of this work.

## 4 Concluding remarks

The application of the FAIR principles to data management has implied a significant (re)evolution over the traditional way scientific data was used, re-used, and shared in the past. Research software is a key component of scientific endeavour, specifically for data-driven domains. The application of the equivalent FAIR principles to research software is a required step to build an integrated ecosystem where research outcomes become fully trustable and reproducible (Lamprecht *et al.* 2020). FAIR for research software can indeed contribute towards choosing the right alternative for having reproducible, well-documented, and interoperable software that is an integral part of the scientific process. We have presented here the first attempt to quantitatively evaluate the FAIRness of research software in Life Sciences at a large scale. This initiative complements other efforts in the field and extends them by applying quality indicators to real research software. Aggregating individual results serve as proof of concept that FAIRsoft indicators allow the automated monitoring of relevant aspects of research software quality in this particular domain. The first generation of indicators represents an opportunity to foster additional interactions across the different initiatives on this particular matter. We have used the Software Observatory section at OpenEBench to collect software quality-related features for over 43 000 tools available at the most popular software registries and repositories in Life Sciences. The collective analysis of such results indicates that research software is moderately accessible and reusable but hardly interoperable. Findability is implicit as data comes from software registries, but other aspects of this principle, like the existence of proper metadata, are also well covered. The analysis of individual indicators like the availability of licences or versioning schemes shows, as expected, a large heterogeneity and the need of setting and popularizing best practices in software engineering among the developer's community (Doerr *et al.* 2019). Perhaps the main limitation of this approach is the automated integration of metadata from different sources at a large scale, as no unambiguous identifier exists for research software in general terms. Remarkably, the most complete indicators come from *ex profeso* developments within OpenEBench, e.g. bioschemas detection, or site accessibility; what points out that the available metadata at registries and repositories may not be enough to fully address this objective, and dedicated developments are necessary for deriving indicators that can be quantified automatically.

This work should be considered an initial effort for having a quantitative overview of the common practices for developing research software in the Life Sciences domain. Proposed indicators can definitely contribute towards the consolidation of the FAIR principles for research software, driven by community efforts, e.g. FAIR4RS, a joint work by the RDA, ReSA, and FORCE11. Indeed, this first generation of indicators should serve to improve automated measurement algorithms as well as to reflect the contribution of specific principles to the four general ones. This work also reveals the need of encouraging developers to annotate software metadata in community-recommended registries and repositories properly. Such efforts will improve the overall research software quality, which can be achieved by setting a clear checklist for developers as proposed for ML/AI models (Walsh *et al.* 2021). The ultimate goal is to contribute to the reproducibility and reliability of scientific outcomes by focusing on one of the key elements for success: Research Software.

Periodic assessment of research software FAIRness would allow researchers to understand the existing and emerging trends regarding the development practices in the community. Such trends can impulse specific actions in establishing best practices for software development in the Life Sciences domain and potentially across other domains facing similar challenges (Schindler *et al.* 2022). The results of this kind of analysis highlight the need for a concerted and transversal effort to ensure the sustainability of scientific endeavours by improving the quality of research software.

## Supplementary Material

btae464_Supplementary_Data
