# Supplementary material for: FAIRsoft—a practical implementation of FAIR principles for research software
Source: Bioinformatics. 2024 Jul 22;40(8):btae464. doi: 10.1093/bioinformatics/btae464 (PMC11330317; doi:10.1093/bioinformatics/btae464)
Supplement: btae464_Supplementary_Data [file btae464_supplementary_data.pdf]

Supplementary Material

Supplementary Material
- Supplementary Figure 1. Findability FAIRsoft indicators.
- Supplementary Figure 2. Accessibility FAIRsoft indicators.
- Supplementary Figure 3. Interoperability FAIRsoft indicators.
- Supplementary Figure 4. Reusability FAIRsoft indicators.
- Supplementary Figure 5: Example of scoring one individual tool (trimAl).
- Supplementary Figure 6. Overview of data retrieval, harmonisation and integration pipeline.
- Supplementary Figure 7. Cumulative distribution of number of sources for individual instances (yellow).
- Supplementary Table 1. Main differences between FAIR4RS and FAIRsoft
- Supplementary Table 2. Primary sources of tools and metadata.
- Supplementary Table 3. Secondary sources of tools metadata.

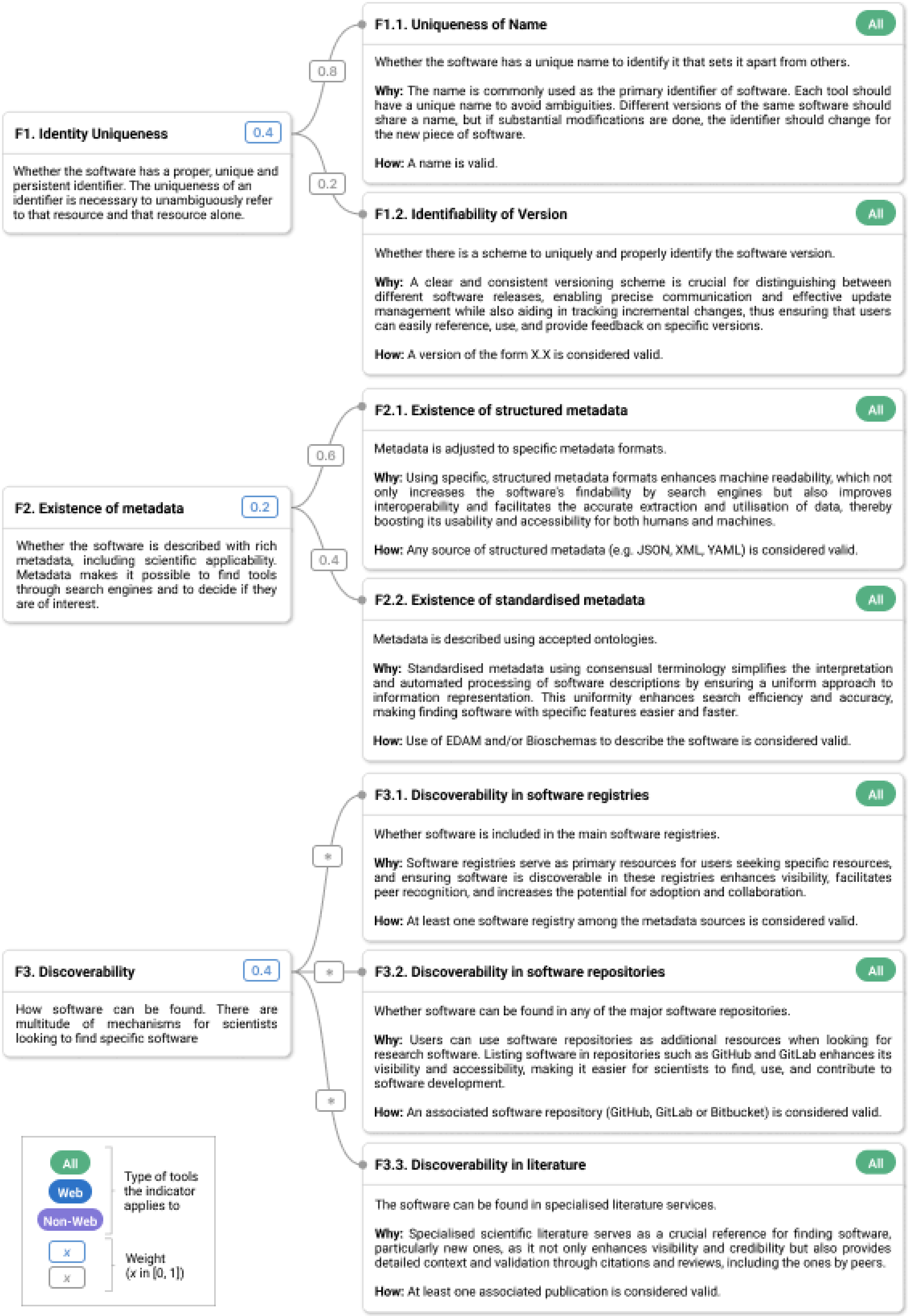

Supplementary Figure 1. Findability *FAIRsoft* indicators.

(*) Scoring of high-level indicator F3 is based on the number of low-level indicators fulfilled: One fulfilled low-level indicator scores 0.7, two fulfilled indicators score 0.85, and the fulfilment of all three indicators scores 1.0

A1. Existence of an available working version (0.7)

Whether it is possible to access/download/build a working version of the software. Being able to access the software as a user is the central aspect of Accessibility.

A1.1. Existence of API or web interface — Web (0.6)

Whether it is possible to access a working version of the software through an API or web interface.

Why: Providing an API or web interface allows remote access with just an internet connection, greatly enhancing the software's usability, facilitating integration with other systems, and enabling a broader range of users to benefit from its features without complex installations.

How: A working URL is considered valid.

A1.2. Existence of downloadable and buildable software working version — Non-Web (0.5)

Whether it is possible to access/download/build a working version of the software including the generation of a software container.

Why: A downloadable and buildable version of the research software significantly increases its freedom of use and usability. It allows users to install and run the software where and as needed, e.g. to use locally and in/as modules.

How: At least one download working link is considered valid.

A1.3. Existence of installation instructions — Non-Web (0.2)

Whether there is a set of instructions and other necessary information that the user can follow to build the software.

Why: Clear installation instructions are essential for ensuring successful software setup, reducing installation and deployment time, minimising potential errors, and enhancing overall user experience.

How: A link explicitly stated as installation instructions or manual, instructions on the web if Bioconductor package, and availability through Galaxy ToolShed are all considered valid.

A1.4. Existence of test data — Web / Non-Web (0.4 / 0.1)

Whether test data is available.

Why: Test data confirms the software's functionality and provides users with practical examples for setting up their own datasets, thereby increasing usability and ensuring robustness.

How: At least one piece of test data is considered valid.

A1.5. Existence of software source code — Non-Web (0.2)

Whether software source code is available.

Why: The availability of source code allows users to compile the software on various operating systems, address installation challenges, manage dependencies, and customise the code to meet specific needs, enhancing adaptability and user control.

How: A link explicitly stated as source code is considered valid.

A2. Software history trackability (0.0)

Whether code and metadata are available even when the software is no longer in use.
To match software provenance with analysed data provenance.

A2.1. Existence of metadata of previous versions in software repositories — All (0.0)

Whether there is available metadata of previous versions.

Why: Maintaining metadata for previous software versions in repositories ensures the traceability of changes and updates, offering insights into the evolution of the software and facilitating the understanding of its historical context and development.

How: Not measured

A2.2. Existence of accessible previous versions of the software — All (0.0)

Whether there are available previous versions.

Why: Accessibility to previous software versions allows users to reproduce analyses with a given software version.

How: Not measured.

⬇ **Additional indicators for Accessibility continue on the next page.**

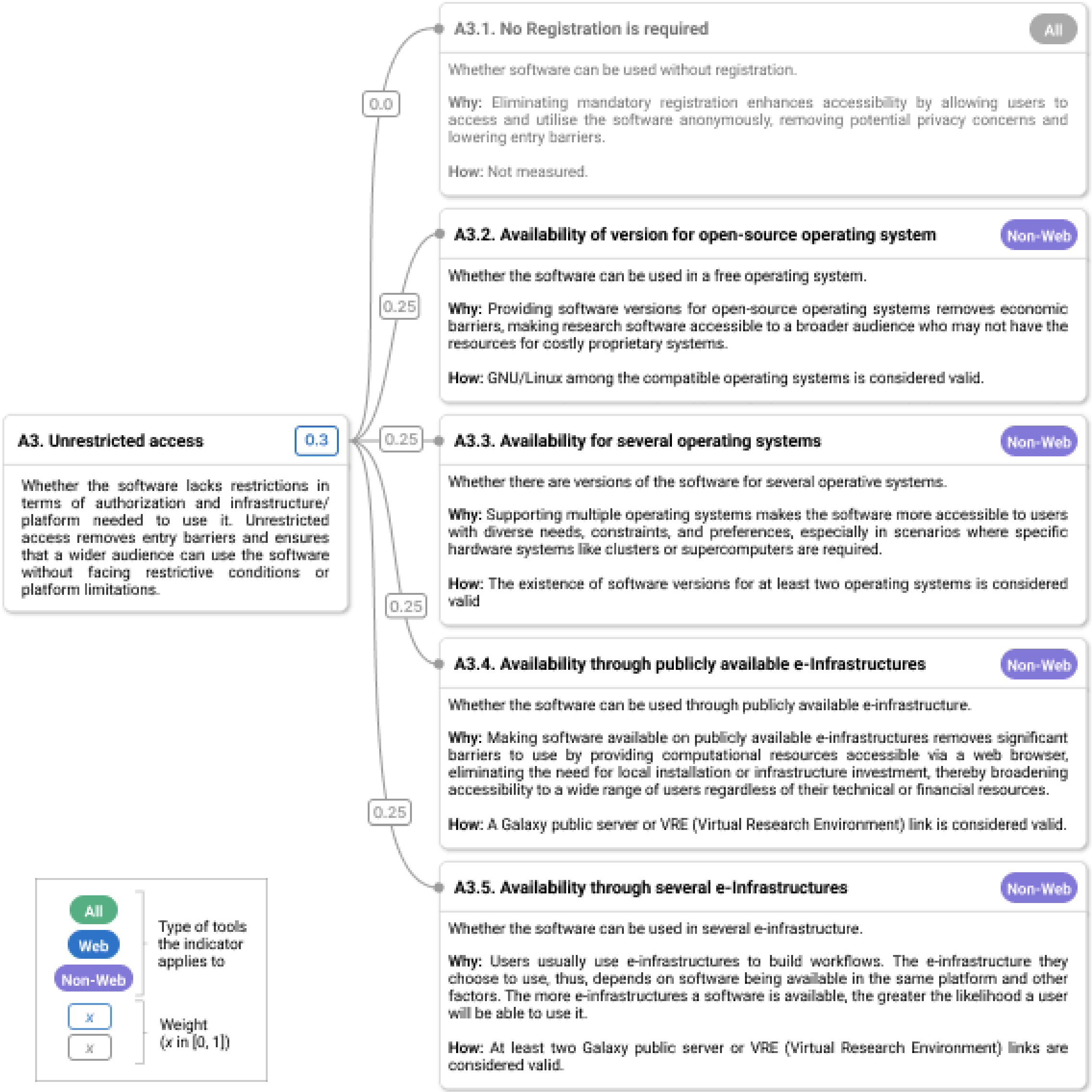

Supplementary Figure 2. Accessibility *FAIRsoft* indicators.

I1. Data Format Standards and Practices — 0.6

Whether the software adheres to data format standards and its operational practices concerning data handling it encompasses standard formats and APIs, the flexibility and verifiability of these formats, and the tracking of data provenance.

Adhering to data format standards and robust data handling practices is critical for ensuring interoperability and reliability across systems, enhancing software integration, maintaining data integrity, and supporting traceability, which boosts user trust and system efficiency.

I1.1. Use of standard data formats — All (0.5)

Whether the input and output data types are formally specified and related to accepted ontologies.

Why: Using standard data formats minimises the need for error-prone and resource-intensive transformations to meet unique software input requirements. It facilitates collective efforts to develop high-quality data management tools, reducing reliance on *ad hoc* data transformations.

How: At least one input or output data format specified in the EDAM format ontology is considered valid.

I1.2. Use of standard API specification framework — Web (0.0)

Whether APIs (REST, libraries) are documented in a standard framework.

Why: Documenting APIs in a standard framework ensures they are universally understandable and easily integrable across different platforms and services.

How: Not measured.

I1.3. Verifiability of data formats — Non-Web (0.3)

Whether input/output data are specified using verifiable schemas.

Why: Verifiability of data formats is crucial as it allows users to automatically confirm that their data complies with accepted standards, ensuring compatibility and readability by other systems using the same format. This verification reduces errors in data processing and enhances overall system interoperability.

How: Any standard formats (I1.1) plus JSON, XML, RDF and XDS are considered valid.

I1.4. Flexibility of data format supported — All (0.2)

Whether the software allows one to choose among various input/output data formats or provides the necessary mechanisms to convert other standard formats into the supported ones.

Why: Supporting various data formats directly in software eliminates the need for data transformations, thereby reducing error points and saving significant time and resources for users, enhancing flexibility and efficiency.

How: At least two input or output data formats are valid.

I1.5. Generation of provenance information — All (0.0)

Whether the software provides provenance information according to accepted standards.

Why: Generating provenance information is crucial for adhering to the FAIR data principles, as it provides detailed documentation that enhances data transparency, traceability, and trustworthiness, thereby supporting improved data management.

How: Not measured.

I2. Software integration — 0.1

Whether software can be easily integrated into workflows or used in connection with other software. Research software is usually used with other software as part of a workflow or any other composition.

I2.1. Existence of API/library version — All (0.5)

Whether the software has an API/library version to be included in users' workflows.

Why: Providing software as modular libraries or well-defined APIs is crucial for seamless integration into larger systems or workflows, ensuring compatibility and functionality across various platforms and environments.

How: The type of the software registered either as *library* or *API* is considered valid.

I2.2. E-infrastructure compatibility — All (0.5)

Whether the software can be deployed in e-infrastructures.

Why: Compatibility with e-infrastructures is critical, as researchers and professionals extensively use these platforms to build data processing pipelines. If software cannot be deployed on these infrastructures, it risks being inaccessible to many potential users, limiting its utility and adoption in essential workflows.

How: Being one of the metadata sources, the availability of the software through the Galaxy Toolshed is considered valid.

⬇ **Additional indicators for Interoperability continue on the next page.**

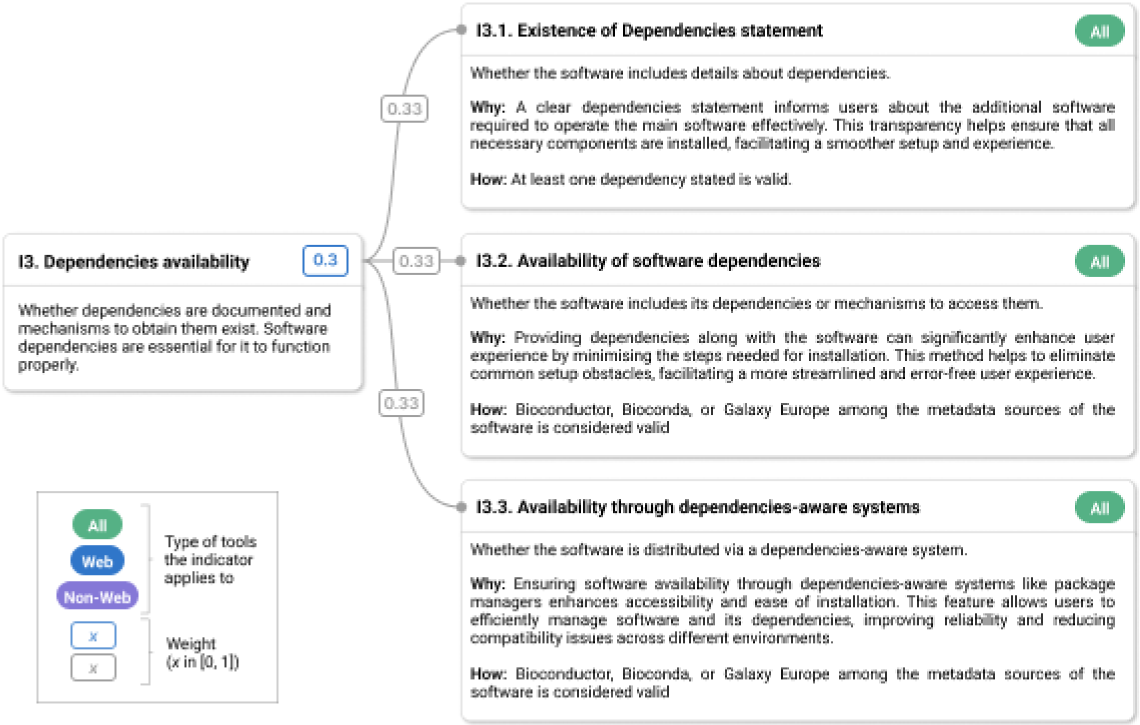

Supplementary Figure 3. Interoperability *FAIRsoft* indicators.

R1. Existence of usage documentation 0.3

Whether software provides adequate usage documentation. Usage documentation such as tutorials, use guides, examples of user cases, and 'how to' allow users to properly and effectively use the software.

1.0

R1.1. Existence of usage guides All

Whether software user guides are provided.

Why: User guides provide comprehensive information for users to understand and use the software. These guides help ensure users can fully exploit the software's capabilities, leading to better user experience and reduced support queries.

How: Any documentation except news, license and terms of use is considered valid.

0.0

R1.2. Existence of usage examples All

Whether examples of use cases are provided.

Why: Usage examples demonstrate practical applications of the software, providing detailed clarity that complements the broader information in user guides and enhances overall understanding.

How: Not measured.

R2. Existence of license and/or terms of use 0.3

Whether a clear and accessible usage license is provided.
Explicit licenses or terms of use are necessary to provide a clear framework for the use of any research software. The lack of a license or terms of use and installation can prevent users, especially from industry and regulated sectors, from installing, executing and using software.

1.0

R2.1. Existence of terms of use Web

Whether terms of use are stated.

Why: Clearly stated terms of use ensure that users are fully aware of the conditions and limitations of software usage before its adoption, promoting transparency and informed decision-making.

How: Terms of use or license are considered valid.

1.0

R2.2. Existence of conditions of use Non-Web

Whether conditions of installation and use are stated.

Why: The existence of clearly defined conditions of use is essential, as it ensures users understand and agree to the terms under which software can be utilised before they begin using it, fostering transparency and compliance.

How: Conditions of use or license are considered valid.

R3. Existence of Contribution Recognition and Governance 0.2

Whether there are policies governing contributor roles and systems in place for acknowledging their contributions ensuring fair and transparent collaboration within software projects.
Clear policies and guidelines on contributing to a software project attract and empower external developers, fostering a collaborative environment.

0.0

R3.1. Existence of the Contributors policy All

Whether a document stating the contributor's policy exists.

Why: A specified contributors policy is crucial because it provides external developers with the necessary guidelines for contributing, facilitating their involvement and enhancing the software's development and improvement.

How: Not measured.

1.0

R3.2. Existence of credit All

Whether credit for contributions is provided.

Why: Credit is essential as it acknowledges contributors' intellectual property rights and ensures their contributions are appropriately recognised under existing regulations and towards the overall research community.

How: Authors are considered valid.

⬇ **Additional indicators for Reproducibility continue on the next page.**

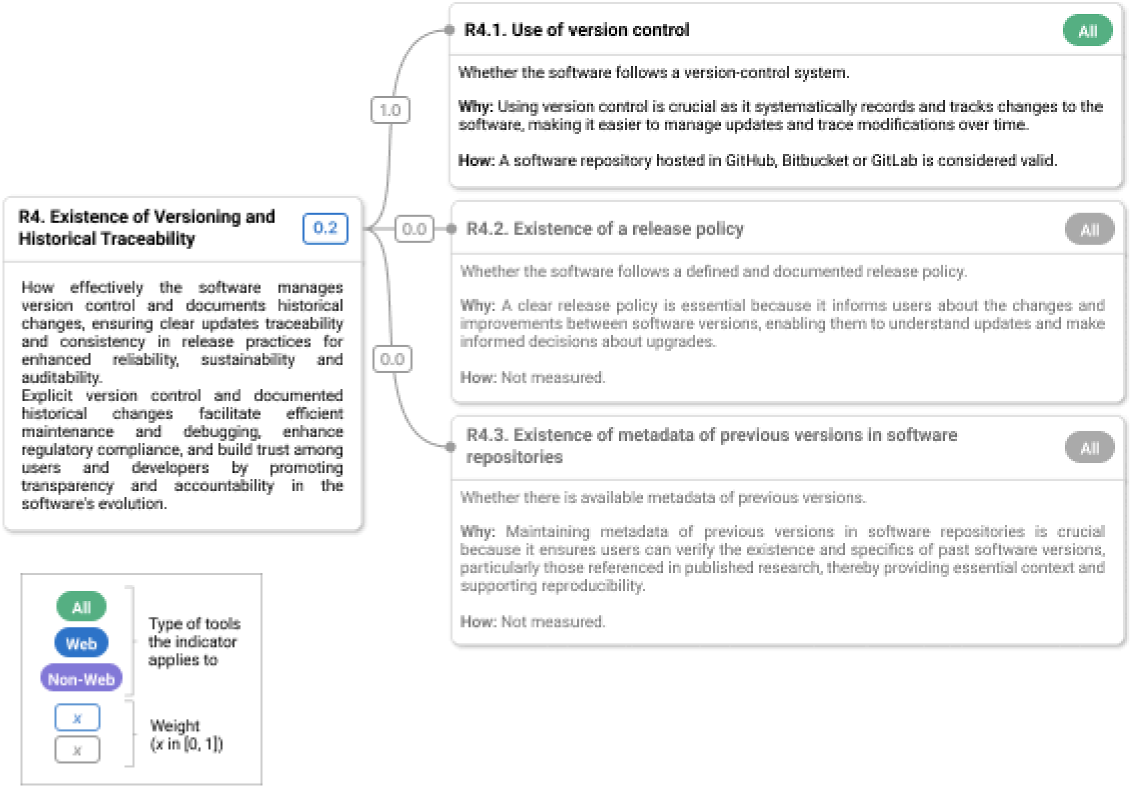

Supplementary Figure 4. Reusability *FAIRsoft* indicators.

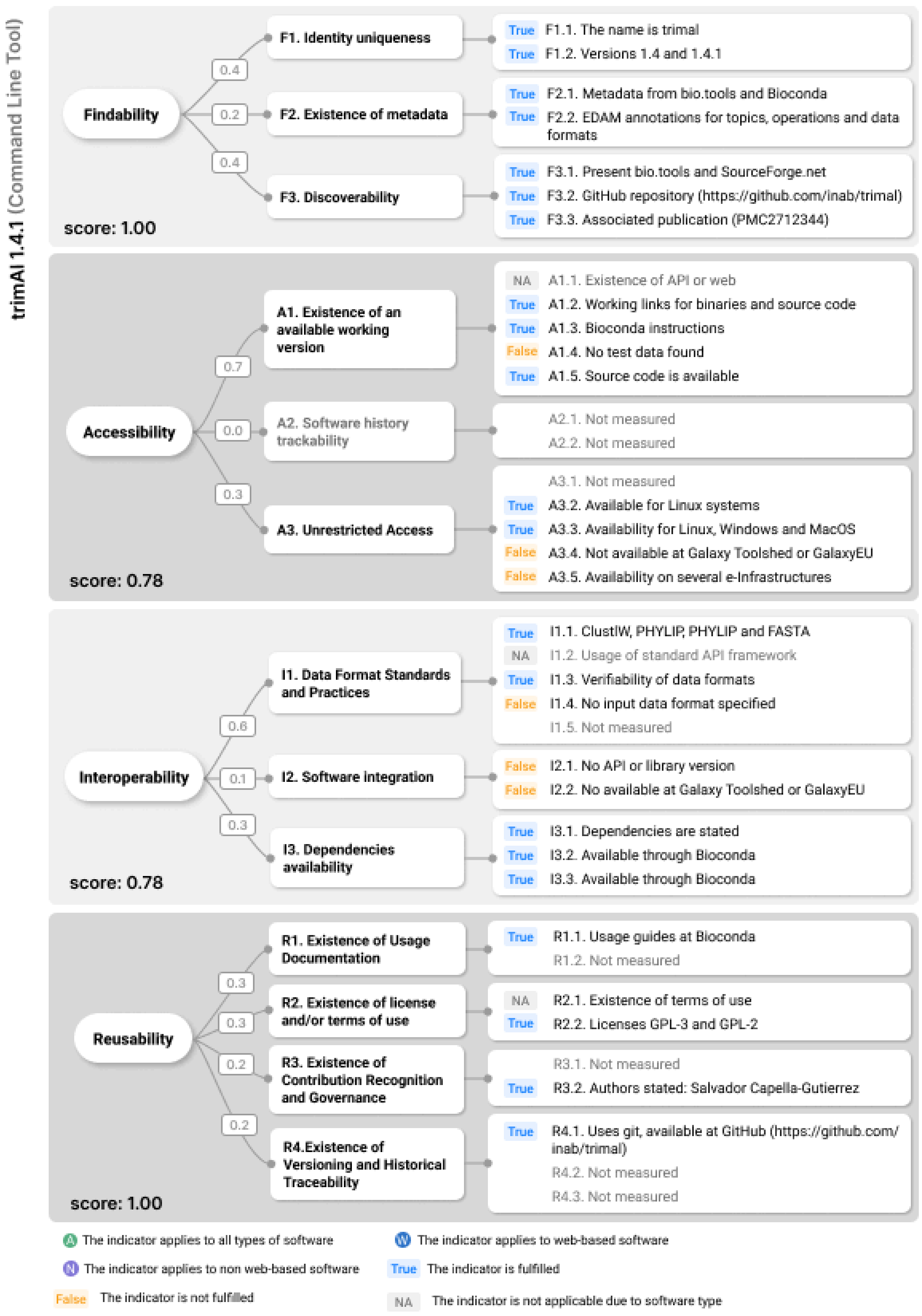

Supplementary Figure 5: Example of scoring one individual tool (trimAl v1.4.1).

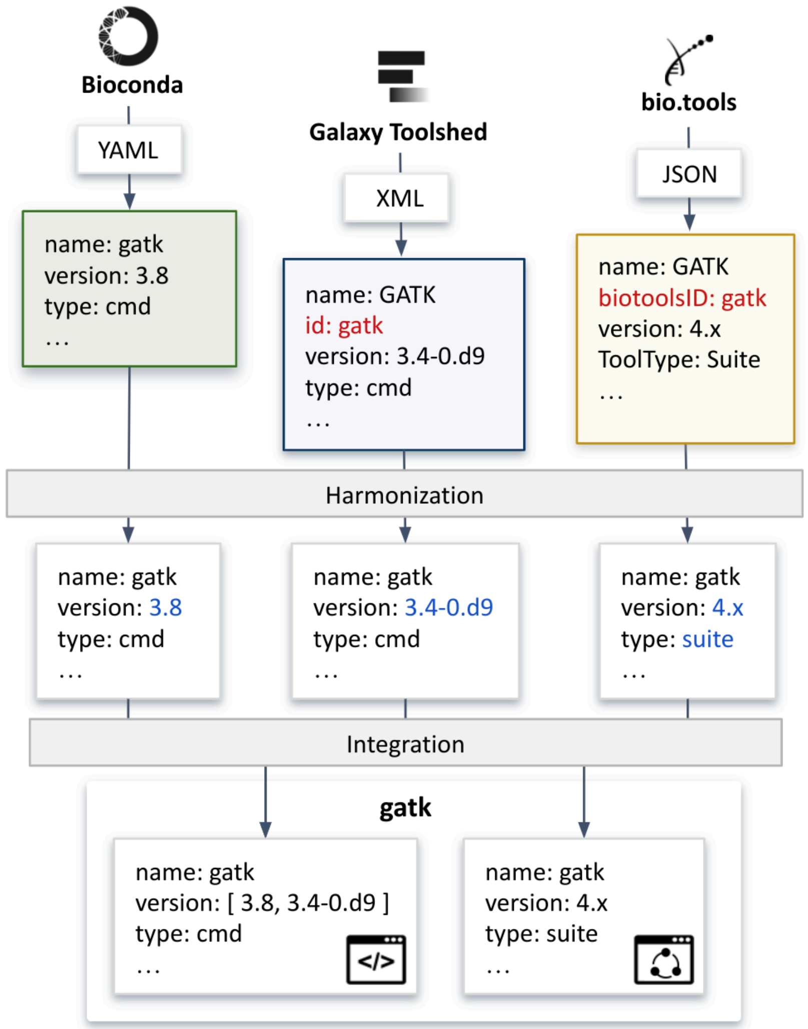

Supplementary Figure 6. Overview of data retrieval, harmonisation and integration pipeline.

Attributes like local 'ID', tool 'name' and/or tool 'label' are concurrent across sources and very likely imply metadata associated with them, actually referring to the same software. We selected one of these attributes for each primary source as the main identifier for consolidation purposes. The attribute selected was the one with which we achieved a more significant overlap with other sources.

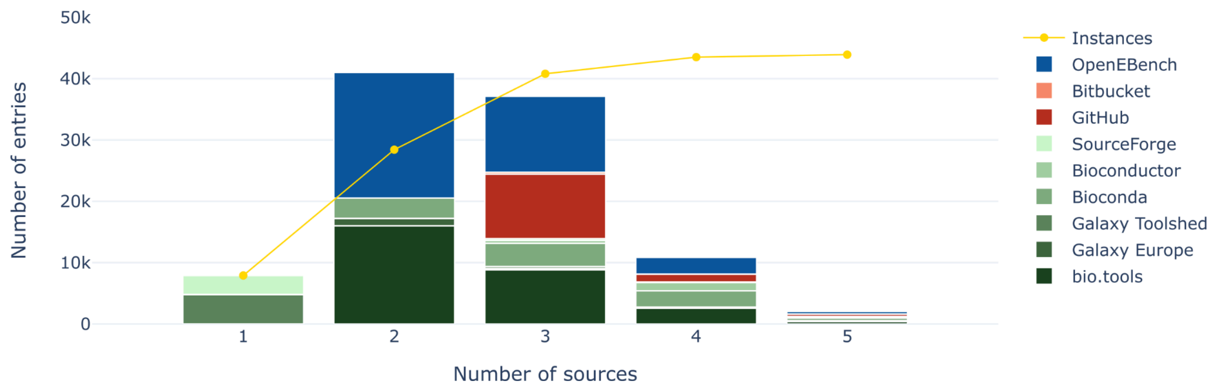

Supplementary Figure 7. Cumulative distribution of number of sources for individual instances (yellow).

Stacked bars represent each source's contribution in terms of the number of metadata entries. Primary sources are coloured in shades of green, and secondary sources are in shades of red.

Almost 18.0% of instances (7,912/43,987) are present in only one source among Galaxy Toolshed and SourceForge. Most of the remaining cases (82%) are found in two sources, bio.tools and OpenEBench, which are the most common combination of provenances.

A)	**FAIR4RS indicators that are not present explicitly in FAIRsoft.**
F1.1	Components of the software representing levels of granularity are assigned distinct identifiers
F3	Metadata clearly and explicitly include the identifier of the software they describe.
A1.2	The protocol allows for an authentication and authorization procedure where necessary.
I2	Software includes qualified references to other objects.
**B)**	**FAIR4RS indicators that are present in FAIRsoft but associated with other principles.**
A1.1	The protocol is open, free, and universally implementable.
R2	Software includes qualified references to other software
R3	The software meets domain-relevant community standards.

Supplementary Table 1. Main differences between FAIR4RS and FAIRsoft

A) Discussion on the FAIR4RS indicators not explicitly present in FAIRsoft.
Addressing the FAIR4RS indicator F1.1, "Components of the software representing levels of granularity are assigned distinct identifiers", we acknowledge its importance in promoting better organisation and traceability within software systems. However, this aspect was not incorporated into our set of indicators due to the vast heterogeneity in software architectures and the practical challenges in automatically measuring such granularity. Often, software granularity is based on arbitrary decisions by developers and requires specific knowledge to infer it, preventing its automated detection and measurement. Generally, when assessing this indicator, developers need to manually consider the structure and identification of software components to enhance their clarity and functionality, especially when dealing with complex systems where such granularity is critical. Our approach focuses on broader, more universally applicable metrics while understanding that some specific, detailed aspects like this may need individual attention beyond the scope of automated assessment.

Our approach to the FAIR4RS indicator F3, "Metadata clearly and explicitly includes the identifier of the software they describe", is inherently built into our methodology. Given that our indicators are designed to be evaluated automatically, primarily through metadata, we assume the presence of an explicit identifier as a prerequisite for our assessments. This assumption allows us to focus on other aspects of metadata quality and accessibility without redundantly verifying the presence of identifiers, which we consider a foundational element already integrated within the metadata we analyse.

The FAIR4RS indicator A1.2, "The protocol allows for an authentication and authorisation procedure where necessary", emphasises the security aspects of the mechanisms to access the systems hosting the research software and not the software itself. When considering FAIRsoft, our indicator A3.1, "No registration is required", prioritises reducing barriers to accessibility for general research software. Unlike sensitive data requiring stringent access controls, general research software can often be made more accessible by minimising compulsory authentication and authorisation procedures. However, we recognise that our indicators, while focusing on accessibility, are not universally applicable and should be adapted based on specific contexts and security needs. Users should assess each indicator's applicability to their situation, especially where security concerns may necessitate deviation from the general guidance to ensure proper infrastructure or data protection and compliance. This flexible interpretation allows for appropriately implementing security measures where they are critically required.

Our FAIRsoft indicators slightly align with the FAIR4RS indicator I2, "Software includes qualified references to other objects", by covering aspects such as ontology annotations for data description (indicator F2.2) and standard data formats (indicator I1.1), but do not explicitly address references to a broader range of digital objects, e.g. datasets or configuration files, or non-digital objects that have a presence in digital systems, e.g. samples, reagents, instruments. This limitation stems from focusing on the foundational aspects of software metadata and usage rather than a comprehensive tracking of all potential digital and non-digital references, which can vary significantly in scope and nature across different software projects.

B) FAIR4RS indicators that are present in FAIRsoft but associated with other principles.
The aspects highlighted by the FAIR4RS indicator A1.1, "The protocol is open, free, and universally implementable", are covered through a combination of our specific criteria. FAIRsoft indicator A1.2, "Existence of downloadable and buildable software working version", focuses on ensuring that users can access and build the software themselves, promoting openness. FAIRsoft indicator A1.1, "Existence of an API or web interface", guarantees a standardised, potentially universal method for interacting with the software, aligning with the Internet protocol's accessible and universally implementable nature. Lastly, the FAIRsoft A3 indicator, "Unrestricted Access", confirms that there are no barriers to accessing the software, which encompasses the free and open criteria of the compared indicator, ensuring broad and equitable usability.

Similarly, the FAIR4RS R2 indicator, "Software includes qualified references to other software", is covered by the FAIRsoft indicators through a comprehensive approach to managing dependencies. The FAIRsoft indicator I3, "Dependencies availability", includes the I3.1 indicator, "Existence of dependencies statement", ensuring all software dependencies are listed; I3.2 indicator, "Availability of software dependencies", confirming that dependencies are accessible; and the I3.3 indicator, "Availability through dependencies-aware systems", which focuses on the use of easy dependencies management through systems that understand and resolve dependencies. This trio of low-level indicators ensures that all software dependencies are not only thoroughly documented but also readily accessible and manageable, aligning with the FAIR4RS's emphasis on qualified and actionable software references.

Finally, the FAIR4RS indicator R3, which focuses on adherence to community standards, must be narrower for a single indicator within the FAIRsoft framework. This is because it encompasses many disparate elements, including using programming languages, testing protocols, file formats, and package managers accepted by the community, among others. However, the FAIRsoft framework does address some of these diverse aspects through specific indicators. For instance, indicator F2.2 emphasises the standardisation of software metadata by adopting community-accepted ontologies. Additionally, indicators I1.1, I1.2, and I1.5 cover using standard data formats, adopting standard API frameworks and generating provenance information.

**Source**	**Structured**	**Retrieval method**	**Data Format**	**Number of instances**
bio.tools	Yes	API	JSON	27,905
Bioconda	Yes	GitHub repository	JSON	10,197
Bioconda	Yes	GitHub recipes repository	YAML	8,611
Bioconda	No	Conda tools	Plain text	9,651
Galaxy Europe	Yes	API	JSON	1,494
Galaxy Toolshed	Yes	Toolshed repository	XML	5,330
Galaxy Toolshed	Yes	API	JSON	3,707
Bioconductor	No	Web	HTML	2,083
SourceForge	No	Web	HTML	3,523

Supplementary Table 2. Primary sources of tools and metadata.

These sources were used to build an initial collection of metadata. Each source required a different retrieval method, resulting in data structuring and formatting across sources. As noted, in the case of Bioconda and Galaxy Toolshed, more than one retrieval method was used to maximise the information extracted from these sources.

Source	Structured	Retrieval	Format	Number of instances
GitHub	Yes	API	JSON	12,184
BitBucket	Yes	API	JSON	387
OpenEBench	Yes	API	JSON	36,045
Pubmed + Europe PMC	Yes	API	JSON	24,621

Supplementary Table 3. Secondary sources of tools metadata.

These sources were used to retrieve metadata about tools already discovered in the primary sources using links and identifiers obtained from them. Repository and publication links obtained from the primary sources were further mined to enrich the initial metadata collection. As with the primary sources, each secondary source required a different retrieval method, resulting in data structuring and formatting across sources.
